# Open transabdominal cisterna chyli ligation for refractory chylothorax: a retrospective case series

**DOI:** 10.1186/s12893-025-03459-7

**Published:** 2025-12-23

**Authors:** Çağatay Çetinkaya, Hasan Fevzi Batırel

**Affiliations:** 1 Department of Thoracic Surgery, School of Medicine, Üsküdar University, İstanbul, Turkey; 2 Department of Thoracic Surgery, School of Medicine, Biruni University, İstanbul, Turkey

**Keywords:** Chylothorax, Cisterna chyli, Transabdominal ligation, Refractory chylous effusion

## Abstract

**Background:**

Chylothorax is a rare but serious condition that can lead to significant morbidity if not effectively managed. While conservative measures and transthoracic duct ligation remain first-line approaches, these can fail in the setting of altered thoracic anatomy, bilateral effusions, or previous thoracic interventions. In such cases, transabdominal ligation of the cisterna chyli offers a direct route to the main lymphatic outflow tract. This study analyzes the results in patients with refractory chylothorax who underwent cisterna chyli ligation through laparotomy.

**Methods:**

In this retrospective case series conducted by a single surgical team, fourteen patients with refractory chylothorax (defined as persistent high-output leakage after failed conservative and/or transthoracic surgical therapy) underwent open transabdominal cisterna chyli ligation between 2011 and 2025. Demographics, etiology, effusion laterality, prior interventions, operative details and outcomes were reviewed. Primary endpoint was resolution; secondary endpoints were hospital stay, complications, recurrence, and mortality.

**Results:**

Median age was 36 years (range 0.4–90), with 9 males. Etiologies were postoperative in 7 (50%), lymphoproliferative disease in 3 (21.4%), and idiopathic in 4 (28.6%). Effusions were right-sided in 5 (35.8%), left in 1 (7.1%), bilateral in 7 (50%), and 1 was intrapericardial. Median postoperative hospital stay was 9 days and median follow-up was 75 months. Nine patients (64.2%) had prior transthoracic surgery. Resolution was achieved in 11 patients (78.6%). Five patients (35.8%) were already intubated in the ICU and remained intubated postoperatively, while the remaining nine were extubated at the end of surgery and returned directly to the ward. Complications included ARDS in one patient and one late recurrence managed successfully with embolization. Three patients had persistent leaks, two due to lymphoma progression and one after esophagectomy.

**Conclusions:**

Transabdominal cisterna chyli ligation appears to be a feasible and effective salvage option for refractory chylothorax, particularly in patients with altered thoracic anatomy or after failed transthoracic interventions. However, conclusions are limited by the small retrospective case series.

## Introduction

Chylothorax is a rare condition characterized by the presence of lymphatic fluid in the pleural space, which can result in significant morbidity and mortality [1]. In adults, the most common cause of chylothorax is iatrogenic injury during surgery—particularly thoracic procedures such as esophagectomy or lung resection—while malignancy, especially lymphoma, is the leading non-traumatic cause. Rare conditions such as lymphangioleiomyomatosis have also been described as etiologic factors [2–4]. Diagnosis is based on the analysis of pleural fluid, which typically appears milky and demonstrates elevated triglyceride levels (> 110 mg/dL) and the presence of chylomicrons [5].

Initial management of chylothorax typically involves conservative measures such as dietary modification with medium-chain triglycerides or total parenteral nutrition, along with drainage of the effusion [5, 6]. Surgical intervention is generally indicated when conservative measures fail—particularly in high-output chylous leaks exceeding approximately 1 L/day for 5–7 days, persistent drainage beyond 2 weeks, or development of nutritional or respiratory compromise [5]. When conservative treatment fails, the current standard surgical approach is thoracic duct ligation—typically via video-assisted thoracoscopic surgery (VATS) through the right hemithorax, with reported success rates exceeding 80% in traumatic cases and 60–87% in non-traumatic chylothorax [7]. Despite its widespread use, thoracic duct ligation via a transthoracic approach can be challenging in cases with distorted anatomy, prior thoracic surgeries, or persistent bilateral chylous effusions, making a transabdominal ligation of the cisterna chyli a valuable alternative [8].

Given the limited number of published cases and the absence of larger series detailing the indications, technical nuances, and clinical outcomes of transabdominal cisterna chyli ligation, the present study aims to: (1) report a consecutive 14-year experience; (2) describe the operative technique with emphasis on key anatomical and technical considerations; and (3) evaluate clinical outcomes, complications, and recurrence patterns in patients with refractory chylothorax.

## Patients and methods

### Study design and setting

This retrospective consecutive case series included all patients who underwent open transabdominal cisterna chyli ligation for refractory chylothorax between January 2011 and May 2025. All operations were performed by the same thoracic surgery team.

### Patient selection and preoperative management

Patients were eligible if they had a confirmed diagnosis of chylothorax based on pleural fluid characteristics (milky appearance, triglycerides > 110 mg/dL, and/or presence of chylomicrons). All patients initially received conservative therapy, including nutritional modification (medium-chain triglycerides or total parenteral nutrition) and continuous drainage. Transabdominal ligation was offered when conservative treatment failed—defined as persistent high-output drainage, prolonged leakage, or clinical deterioration—or when a prior transthoracic thoracic duct ligation or interventional attempt was unsuccessful. Preoperative evaluation included imaging to define laterality, etiology, and anatomical considerations, as summarized in Table 1.

**Table 1:** Baseline characteristics of patients undergoing open transabdominal cisterna chyli ligation

Variable	Value
Number of patients, *n*	14
Age, median (range)	36 (0.4–90)
Sex, *n* (%)	
Male:	9 (64.2%)
Female:	5 (35.8%)
Etiology, *n* (%)	
Postoperative:	7 (50%)
Lymphoproliferative disease:	3 (21.4%)
Idiopathic:	4 (28.6%)
Side of chylous effusion, *n* (%)	
Right:	5 (35.8%)
Left:	1 (7.1%)
Bilateral:	7 (50%)
Isolated pericardial:	1 (7.1%)
Ventilatory status at time of surgery, *n* (%)	
Intubated:	5 (35.8%)
Non intubated:	9 (64.2%)
Prior interventions, *n* (%)	
Prior transthoracic ligation or surgical attempt:	9 (64.2%)
None:	5 (35.8%)

### Surgical technique

**Fig. 1:**
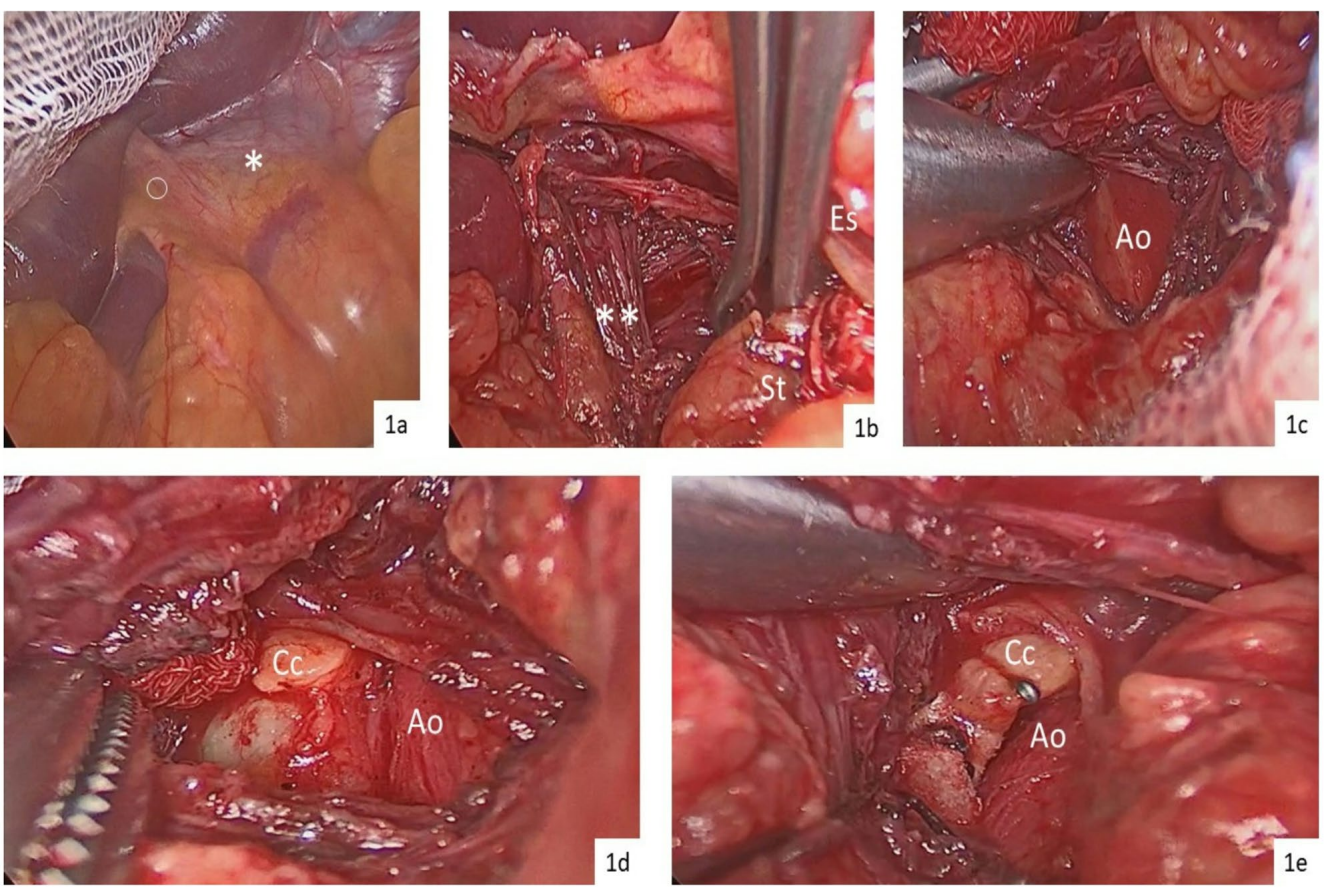
Intraoperative steps of open transabdominal cisterna chyli ligation. **a** Initial view after laparotomy, before division of the gastrohepatic ligament. **b** Retraction of the stomach to the left and the right crus to the right, revealing the thin muscular layer overlying the aorta at the base of the retrocrural space. **c** Clear exposure of the aorta following division of the overlying muscle fibers. **d** Delicate dissection between the aorta and cisterna chyli to fully expose the lymphatic structure. **e** Final appearance after ligation and clipping of the cisterna chyli. Abbreviations: o: Hepatic accessory nerve; *: Gastrohepatic ligament; ** Right crus; St: Stomach; Es, Esophagus; Ao: Aorta; Cc, Cisterna chyli

All procedures were performed through an upper midline laparotomy under general anesthesia with the patient in a supine position. The initial intraoperative view before division of the gastrohepatic ligament is shown in Fig. 1a. The gastrohepatic ligament was divided preserving the accessory hepatic nerve. Care is taken not to injury an aberrant left hepatic artery from the left gastric trunk which was seen in one patient in this series. This maneuver exposes the diaphragmatic crura and the median arcuate ligament (Fig. 1b).

The esophagus was gently elevated superiorly, and the median arcuate ligament—often hypertrophic and moderately vascular in newborns and muscular patients—was divided vertically to access the retrocrural space. In some cases, approximately 1 cm of overlying muscular fibers was divided using an energy device to reach the aortic adventitia and the cisterna chyli (Fig. 1c).

The descending aorta was then retracted to the left, enabling identification of the antevertebral lymphatic tissue between the aorta and the right diaphragmatic crus, where the cisterna chyli typically lies (Fig. 1d). Mass ligation of this lymphatic tissue was performed using two pledgeted non-absorbable No. 0 silk sutures placed approximately 1 cm apart, followed by application of clips on the superior lymphatic trunk (Fig. 1e).

### Data collection and outcome definitions

Data were retrospectively extracted from electronic medical records, including demographics, etiology, effusion laterality, ventilatory status, prior interventions, and perioperative parameters. The primary endpoint was complete resolution of the chylous leak without the need for additional surgical or interventional treatment. Secondary endpoints included postoperative extubation status, time to leak cessation, duration of chest drainage, hospital length of stay, complications, recurrence, and mortality. Complications were defined as any deviation from the expected postoperative course, and recurrence was defined as reaccumulation of chylous fluid requiring therapeutic intervention after initial resolution. Follow-up assessments were performed routinely at postoperative week 1, month 1, and subsequently at 3-month intervals. Clinical evaluation and chest radiography were used at each visit to monitor for recurrence or late complications, as summarized in Table 2.

**Table 2:** Operative and postoperative outcomes following open transabdominal cisterna chyli ligation

Variable	Value
Success of transabdominal ligation, *n* (%)	
Successful:	11 (78.6%)
Unsuccessful:	3 (21.4%)
ICU care, *n* (%)	
Postoperative ICU care required:	5 (35.8%)
No postoperative ICU required:	9 (64.2%)
Postoperative hospital stay, median (range), days	9 (5–35)
Complications, *n* (%)	
ARDS:	1 (7.1%)
None:	10 (92.9%)
Late Recurrence, *n* (%)	
Yes:	1 (7.1%)
No:	10 (92.9%)
Mortality, *n* (%)	1 (7.1%)

### Statistical analyses

Statistical analysis was descriptive. Continuous variables were summarized as median and range, and categorical variables as absolute numbers and percentages. No inferential statistical testing was performed due to the small sample size and retrospective case-series design.

## Results

**Fig. 2:**
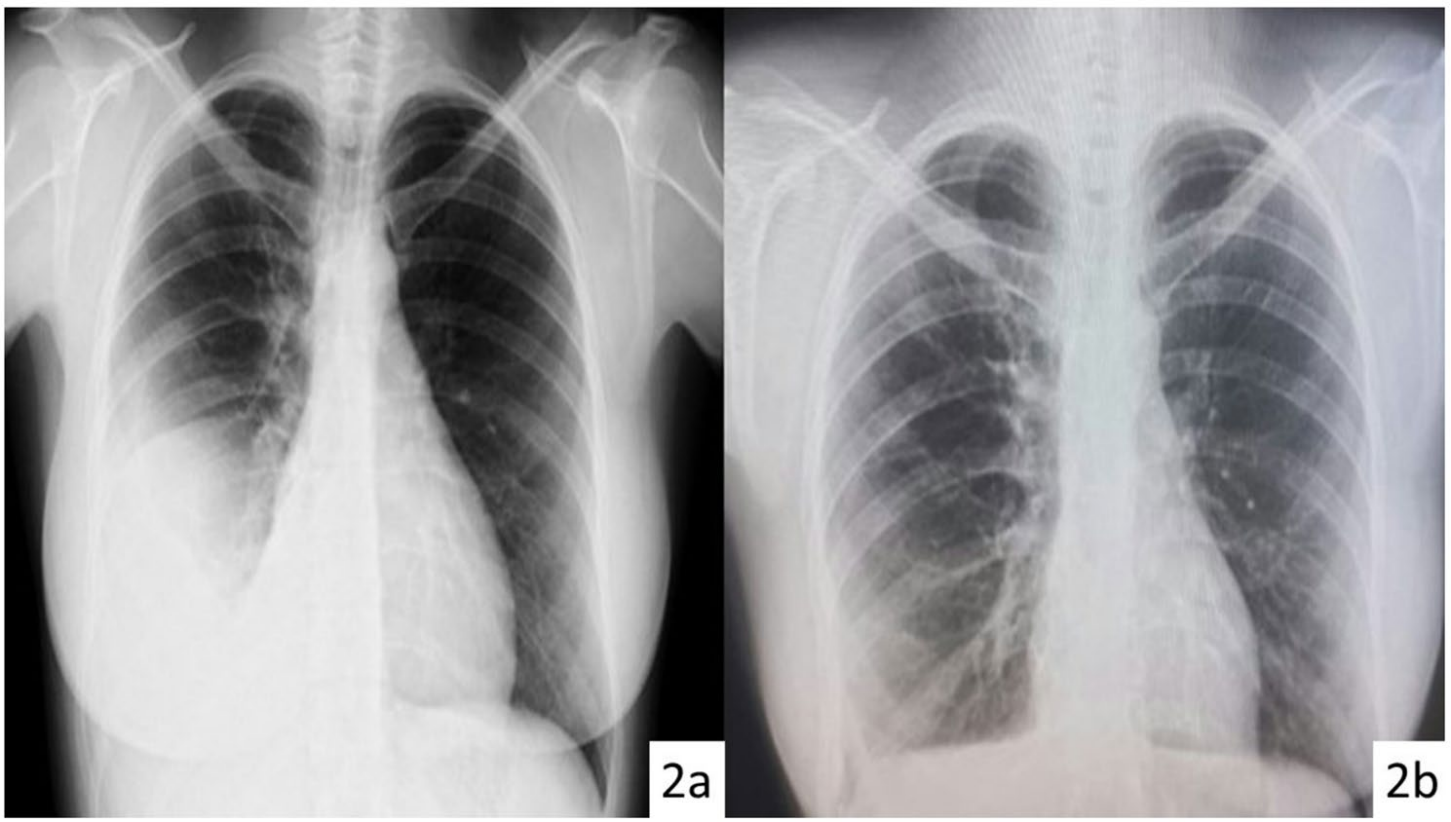
Preoperative (**a**) and postoperative (**b**) chest radiographs of a 29-year-old patient with persistent chylothorax after failed right-sided thoracic duct ligation via thoracotomy. The patient subsequently underwent transabdominal cisterna chyli ligation, resulting in spontaneous resolution of the effusion by the third postoperative month

A total of 14 patients underwent transabdominal cisterna chyli ligation between 2011 and 2025. The median age was 36 years (range, 0.4–90 years), and 9 patients were male. The underlying causes of chylothorax were postoperative in 7 patients (50.0%)—including 3 esophagectomies, 1 lung resection, 1 mediastinal tumor resection, 1 mediastinitis drainage, and 1 single-ventricle repair—while 3 patients (21.4%) had lymphoproliferative disease and 4 (28.6%) were idiopathic. Pleural chylous accumulation within the thoracic cavity was located in the right hemithorax in 5 patients (35.8%), in the left hemithorax in 1 patient (7.1%), and bilaterally in 7 patients (50.0%). In addition, 1 patient (7.1%) had isolated intrapericardial chylous accumulation without pleural involvement. At the time of surgery, 5 patients (35.8%) were under mechanical ventilation. Nine patients (64.2%) had previously undergone at least one transthoracic surgical attempt to control the chylous leak. Transabdominal cisterna chyli ligation resulted in successful resolution of chylous leak in 11 out of 14 patients (78.6%). Among the 14 patients, nine had an indwelling chest tube at the time of surgery. In three of these patients (two with lymphoma and one post-esophagectomy), chylous drainage persisted despite transabdominal ligation. In the remaining six patients, complete cessation of chyle leakage was achieved, and chest tubes were subsequently removed after a median of 5 days (range 2–8 days). Two patients had no chest tube before surgery and did not require postoperative drainage. Both demonstrated partial radiographic improvement prior to discharge, and complete resolution of the pleural effusion was confirmed at the one-month follow-up. An example of complete resolution following transabdominal ligation is demonstrated in Fig. 2.

Five patients (35.8%) were already intubated in the ICU preoperatively and remained intubated postoperatively for continued critical care. The remaining nine patients were operated on from the ward and were transferred back to the ward extubated after surgery without requiring postoperative ICU stay.

Surgical intervention was unsuccessful in 3 patients: 2 with underlying lymphoma, and 1 post-esophagectomy patient whose leak persisted despite a partial reduction in drainage. One of the post-esophagectomy patients died due to acute respiratory distress syndrome (ARDS), although the chylous leak had ceased following surgery. One patient developed a late recurrence two years after surgery, which was successfully treated with percutaneous embolization.

## Discussion

Chylothorax remains a rare and challenging clinical condition, and its optimal management continues to evolve, particularly in patients who do not respond to conservative therapy or transthoracic surgical intervention. In this context, our series adds meaningful real-world data on the role of open transabdominal cisterna chyli ligation as a salvage approach. Below, we discuss how our findings relate to previous reports, the anatomical and technical rationale for this approach, and its potential advantages, limitations, and clinical implications.

### Comparison with previous reports

Although thoracic duct ligation via a transthoracic approach—especially through VATS—has become the standard surgical treatment in patients unresponsive to conservative management, its success can be limited in cases with altered anatomy, bilateral effusion, or previous thoracic interventions. In such cases, alternative approaches including percutaneous thoracic duct embolization have been shown to be effective [9, 10].

In selected cases where transthoracic approaches are not feasible or have previously failed, the transabdominal route provides a viable alternative for accessing and ligating the cisterna chyli. Recent reports have described successful treatment of refractory chylothorax using laparoscopic cisterna chyli ligation in small patient series [8], while additional case reports have demonstrated technical feasibility through alternative minimally invasive routes, including transhiatal and robotic-assisted approaches [11–13].

Given the very limited number of published cases utilizing a transabdominal approach, our series offers additional real-world data by presenting a consecutive cohort treated over a 14-year period. Although modest in size, this experience expands the available evidence by demonstrating that cisterna chyli ligation can be performed across a clinically heterogeneous group, including postoperative, malignant, and idiopathic etiologies. Furthermore, the findings suggest that patients with refractory or high-output chylothorax, those with prior thoracic surgery, or individuals in whom transthoracic access or embolization is not feasible may particularly benefit from consideration of an abdominal approach.

### Factors associated with unsuccessful outcomes

Three patients in our series did not achieve complete resolution of the chylous leak, and all had challenging underlying conditions (two with lymphoma and one with post-esophagectomy chylothorax). Previous reports have consistently shown lower success rates of thoracic duct or cisterna chyli ligation in malignant chylothorax compared with postoperative or traumatic etiologies, attributed to diffuse lymphatic involvement and persistently elevated lymphatic flow [1, 5, 6]. Likewise, several case-based publications have documented persistent leakage after esophagectomy despite anatomically adequate ligation, often attributed to anatomical variation or multiple lymphatic channels outside the standard ligation field [8, 14]. These findings suggest that the underlying disease process rather than the transabdominal approach itself likely contributed to the failures observed in our cohort.

In addition, technical exposure was markedly limited in these patients. In the two lymphoma cases, bulky vascular lymph nodes hindered visualization of the cisterna chyli, while in the post-esophagectomy patient, a large stomach conduit covering the median arcuate ligament restricted access to the lymphatic outflow tract. These factors likely resulted in suboptimal technical ligation, compounding the difficulty imposed by the underlying disease. Taken together, these observations suggest that the disease process and technical exposure limitations—rather than the transabdominal approach itself—likely contributed to the unsuccessful outcomes in our cohort.

Recent case-based literature has further expanded the spectrum of mechanisms leading to thoracic duct or cisterna chyli disruption, beyond classical postoperative and malignant etiologies. Unusual non-traumatic presentations, such as delayed spontaneous chylothorax following yoga practice, underline how even minor thoracoabdominal strain may precipitate clinically significant lymphatic leaks in predisposed individuals [15]. Likewise, refractory chylothorax secondary to sizeable vascular malformations of the azygos system has been described, requiring highly individualized multimodal treatment strategies [16]. These observations support the concept that diverse and sometimes subtle anatomical or biomechanical factors can underlie chylous effusions, reinforcing the need for tailored management algorithms and careful preoperative mapping of the thoracic duct–cisterna chyli axis when planning salvage procedures such as transabdominal ligation.

### Technical considerations and anatomical rationale

Although the transabdominal approach provides direct access to the cisterna chyli and may offer higher success rates in selected patients—particularly after failed transthoracic interventions or in bilateral effusions—it remains a technically demanding procedure due to anatomical variability and the deep retroperitoneal location of the target structure. Anatomically, the cisterna chyli is most often located anterior to the L1–L2 vertebral bodies, posterior to the right diaphragmatic crus, and between the abdominal aorta and inferior vena cava. Cadaveric studies have demonstrated its presence in approximately 83% of individuals, with some positional variations.

This relatively consistent retrocrural location makes the cisterna chyli more accessible through a transabdominal route than through transthoracic approaches, particularly when performed via open laparotomy [17]. In addition, the ability to obtain direct proximal control of the lymphatic inflow at the origin of the thoracic duct may help explain the favorable success rates observed in selected refractory cases. However, precise identification of the retrocrural lymphatic tissue is essential, as unrecognized anatomical variants or collateral channels may contribute to persistent leakage if not adequately controlled.

### Advantages and limitations of the transabdominal approach

One of the principal advantages of the transabdominal approach is its ability to provide access to the most inferior portion of the lymphatic pathway, allowing ligation at the level of the cisterna chyli—an anatomical region that may be beyond the reach of transthoracic techniques. By obtaining proximal control of lymphatic inflow at this level, the procedure offers a physiologically favorable point of intervention, which may help explain the higher success rates observed in selected refractory cases. Furthermore, transthoracic ligation may be limited by anatomical variability or unrecognized collateral branches within the thorax, whereas the retrocrural location of the cisterna chyli is relatively consistent and therefore amenable to exposure via a transabdominal route.

In clinical practice, this approach may be most relevant for a subset of patients in whom transthoracic access is challenging or has previously failed. These include individuals with refractory or high-output chylothorax, patients with prior thoracic surgery or dense pleural adhesions, and those who are not candidates for percutaneous thoracic duct embolization due to anatomical constraints or unsuccessful prior attempts. In such scenarios, the ability to access the cisterna chyli from the abdomen provides an alternative route when conventional interventions are limited.

Among these advantages, this anatomical position allows proximal control of lymphatic flow and may contribute to improved outcomes in selected patients. In contrast, transthoracic ligation may fail due to anatomical variability or unrecognized collateral lymphatic branches within the thorax.

However, transabdominal cisterna chyli ligation is inherently an open procedure, and although laparoscopic approaches have been reported, they remain technically challenging because of the posterior location of the cisterna chyli and limited retrocrural exposure. Adjunctive tools such as indocyanine green (ICG) fluorescence may improve visualization of lymphatic channels during minimally invasive exploration [18], but are not widely available and have not been standardized. In pediatric and neonatal patients, access may be further complicated by hypertrophic crural musculature and a smaller retrocrural working space.

Moreover, due to the rarity of this indication, many surgeons are not familiar with the detailed retroperitoneal anatomy required for this approach.

### Study limitations

This study has several limitations. The retrospective design and the small sample size inherently restrict the strength of the conclusions and limit the generalizability of the findings. In addition, the cohort included patients with diverse underlying etiologies of chylothorax, which introduces clinical heterogeneity and may influence both the presentation and response to treatment. Although our results provide insight into the feasibility of transabdominal cisterna chyli ligation in refractory cases, the lack of standardized preoperative imaging across all patients limits the ability to fully characterize anatomical variations that might impact surgical success.

A further limitation is the absence of long-term, standardized lymphatic follow-up. While no patient exhibited clinically apparent late complications during routine postoperative evaluations, dedicated imaging modalities such as MR lymphangiography or lymphoscintigraphy were not systematically performed, and therefore subclinical lymphatic dysfunction cannot be excluded. Additionally, the study lacks a comparison group, particularly against contemporary interventional radiology–based treatments such as thoracic duct embolization, which has become an important alternative in many centers. As a result, the relative effectiveness of the surgical approach presented here cannot be directly assessed.

An important strength of our series is its heterogeneity—including postoperative, malignant, and idiopathic etiologies—which highlights the broader applicability of the transabdominal approach across diverse mechanisms of chylothorax.

### Future directions

The technical complexity of transabdominal cisterna chyli ligation highlights the need for structured anatomical training, and the development of cadaveric or simulation-based models may help improve familiarity with central lymphatic anatomy. Future research comparing this approach with minimally invasive or interventional radiology–based techniques could clarify where each modality fits within the treatment algorithm for refractory chylothorax.

Although our experience adds to the limited literature, these findings should be considered preliminary given the small retrospective cohort. Well-designed multicenter studies will be essential to validate outcomes and refine patient selection for this uncommon but challenging condition.

## Conclusion

In this small retrospective series, transabdominal ligation of the cisterna chyli appeared to be a feasible salvage option for selected patients with refractory chylothorax who did not respond to conservative or transthoracic interventions. Although technically demanding, the approach provides direct access to the proximal lymphatic outflow tract and may be particularly useful in cases with altered thoracic anatomy or prior surgical scarring. While our findings support the potential role of this rarely performed technique, the conclusions remain limited by the sample size and study design. Larger multicenter studies and longer follow-up are needed to better define indications, long-term outcomes, and the role of minimally invasive variants of this approach.

## Data Availability

The datasets used and/or analysed during the current study are available from the corresponding author on reasonable request.

## Abbreviations

Ao: Aorta
ARDS: Acute respiratory distress syndrome
Cc: Cisterna chyli
Es: Esophagus
GHL: Gastrohepatic ligament
ICG: Indocyanine green
St: Stomach
VATS: Video-assisted thoracoscopic surgery
